# Notes from Past Meetings of the Lippe Society

**Published:** 1892-11

**Authors:** 


### NOTES FROM PAST MEETINGS OF THE LIPPE SOCIETY.

Dr. Fellger described a case of gonorrhœa in which the patient suffered most excruciating pain day and night. His eyes were sunken, he had had no sleep for three days. Pain along the urethra into the bladder, with constant desire to urinate demanded Cantharis. This remedy was given in the 200th, and in a few hours he was able to sleep and all pain disappeared. At another meeting Dr. Lippe read a paper entitled : "Our Materia Medica, how to Preserve and Augment it." The subject of the paper was opposition to the views of those who wish to belittle the materia medica.

Dr. Fellger said : Before we begin to condense the materia medica we should condense pathology. It is perfectly nonsensical to speak of condensing symptoms ; they cannot be condensed, and yet our materia medica consists only of symptoms. The difficulty is, we often find symptoms in patients that cannot be found in provings. Many valuable symptoms have been lost because they were thought to be of no value. Every symptom is of importance.

Dr. Carleton Smith : The very ones who are howling for condensation are always howling for new remedies. They do not know the old remedies sufficiently well to use them properly.

Dr. Fellger : An old-school physician once said, "For all the diseases we cannot cure we have the greatest number of remedies. This proves we cannot cure them ; therefore we are always looking for others."

